# Galectin-9 expression clinically associated with mature dendritic cells infiltration and T cell immune response in colorectal cancer

**DOI:** 10.1186/s12885-022-10435-4

**Published:** 2022-12-16

**Authors:** Yang Wang, Ruizhi Zheng, Yanhui Zhang, Yuhong Guo, Zhenzhen Hui, Peijing Wang, Yan Sun

**Affiliations:** 1 Tianjin Medical University Cancer Institute and Hospital, National Clinical Research Center for Cancer, Tianjin, 300060 China; 2 Tianjin’s Clinical Research Center for Cancer, Tianjin, 300060 China; 3 Key Laboratory of Cancer Immunology and Biotherapy, Tianjin, 300060 China; 4 Department of Pathology, Tianjin Cancer Institute and Hospital, Tianjin Medical University, Huanhu West Road, Hexi District, Tianjin, 300060 China; 5 The Third Central Clinical College of Tianjin Medical University, Tianjin, 300170 China; 6 Department of Neurology, The Third Central Hospital of Tianjin, Tianjin, 300170 China; 7 Department of Biotherapy, Tianjin Medical University Cancer Institute and Hospital, Tianjin, 300060 China

**Keywords:** Galectin-9, Colorectal cancer, Mismatch repair-proficient, Dendritic cells, Immunotherapy

## Abstract

**Background:**

Galectin-9 is a member of the galectin family and has been reported to have a tumor-promoting or antitumor effect in response to the immune microenvironment. However, the immunomodulatory effect of galectin-9 in colorectal cancer (CRC) remains unclear. The antigen presentation and antitumor immune effects of galectin-9 in CRC were examined in this study.

**Methods:**

The expression of galectin-9, dendritic cell markers (CD208 and CD1a), T-cell markers (CD3 and CD8) and mismatch repair proteins (MLH1, PMS2, MSH2, and MSH6) was assessed using immunohistochemistry in CRC samples. The correlation between galectin-9 and immune cells or immunomodulatory factors was also evaluated via multiple gene expression databases.

**Results:**

The level of galectin-9 was decreased in mismatch repair-proficient patients compared with mismatch repair-deficient patients (*p* = 0.0335). GSEA showed that the regulatory mechanism of galectin-9 in CRC was related to a variety of immune pathways. Galectin-9 expression was strongly correlated with immune cell infiltration and immunomodulators (all *p* < 0.0001). In the relationship between galectin-9 expression and the infiltration of DCs, there was a negative correlation in CD1a + immature DCs (*R* = -0.263, *p* = 0.042). A strong positive correlation was observed in CD208 + mature DCs (*R* = 0.391, *p* < 0.01). Patients with high galectin-9 expression also exhibited abundant CD8 + T-cell and CD3 + T-cell infiltration.

**Conclusion:**

Collectively, our findings provide evidence that galectin-9 may increase the antitumor immune response of patients with CRC. DCs play an important role in galectin-9-mediated antitumor immune responses, which provides further insight into the development of immunotherapy.

**Supplementary Information:**

The online version contains supplementary material available at 10.1186/s12885-022-10435-4.

## Introduction

Colorectal cancer (CRC) remains one of the world's most common and deadly cancers and presents serious threats to global health [1]. Immune checkpoint inhibitors (ICIs), especially anti-PD-L1 therapy, have rapidly changed the treatment paradigm for multiple cancer types. Unfortunately, only patients with mismatch repair-deficient (dMMR) tumors can benefit from this treatment strategy in CRC [2]. These tumors were estimated to comprise 15% to 20% of total CRC cases and approximately 4% of stage IV CRCs [3]. Therefore, patients with mismatch repair proficiency (pMMR) (approximately 90%) present a clinical challenge, with significant resistance to current immunotherapies (response rate of only 5%–10%) [4, 5]. Thus, it is an urgent task to better understand the molecular basis of CRC development and identify new prognostic markers and therapeutic targets for future individualized treatment strategies.

Extensive tumor inflammation improves the response of patients to ICIs, which is reflected by rich infiltrating T cells and interferon-γ (IFNγ). Many tumors, however, have primary and acquired resistance through multiple mechanisms that lead to immunosuppression [6]. One such mechanism is the impairment of antigen presentation [7, 8]. To elicit an effective antitumor response, antigen presentation has to be successful at two distinct events: first, professional antigen-presenting cells (mostly dendritic cells (DCs)) uptake cancer neoantigens and cross-present them to naive CD8 + T cells. Second, the neoantigens have to be directly presented by tumor cells for recognition and killing by primed CD8 + T cells [9]. The abundance of DCs in the tumor microenvironment (TME) is associated with T cell infiltration, better overall survival (OS) and response to ICIs in cancer patients [10]. Various tumor-derived factors mediate DC function, and some interactions are being investigated.

Galectin-9 was originally described as an eosinophil chemoattractant and a potent immunomodulator [11]. Some previous studies suggest that tumors can lead to immunosuppression through the Tim-3/galectin-9 pathway. However, clinical trials using inhibition of Tim-3/galectin-9 in patients did not reveal improved efficacy [12], highlighting the incomplete understanding of the role of these molecules. Galectin-9 both promotes and inhibits tumor activity, depending on its interactions with its ligands on T cells, antigen-presenting cells or tumor cells, the experimental conditions, and the balance of immunity in specific TMEs [13]. Based on preliminary research, we found that galectin-9 expression was reduced and was associated with poor prognosis in CRC patients [14], suggesting that this molecule plays an antitumor role in CRC. However, no study has assessed the differences in the expression levels of galectin-9-based MMR expression. Furthermore, we found that it is also involved in regulating NK cell recruitment [14]. NK cells are the main producers of FLT3 L in the TME, and FLT3 L promotes the development, proliferation and survival of DCs [15]. NK cells can promote the recruitment of DCs through FLT3 L to exert antitumor immunity [16]. Based on the above results, we speculate that galectin-9 may play a crucial role in regulating the function of DCs, which in turn affects the antigen presentation function.

In this study, we investigated the basic functions of galectin-9 and studied the relationship between galectin-9 and a variety of tumor-infiltrating immune cells as well as tumor immunity status via comprehensive bioinformatics analyses. There was a positive correlation between galectin-9 expression and mature DC and T-cell infiltration. In addition, galectin-9 expression was upregulated in dMMR tumors and the right colon. These results provide new insights into the functional role of galectin-9 in CRC.

## Methods

### Patients and tissues

Human tumor and paired adjacent noncancerous tissue sections from 139 CRC patients at the Tianjin Cancer Institute and Hospital were analyzed between January 2016 and December 2019. All patients were confirmed by histology. This study was approved by the Institutional Review Board of Tianjin Medical University Cancer Hospital, and all patients in the study provided written consent.

### Immunohistochemistry

An immunohistochemical (IHC) study on galectin-9 (54330 s, Cell Signaling Technology (CST); 1:500), mouse anti-human CD208 (LAMP3) antibody (to define mDCs; DDX0191P-100; Novusbio USA; 1:10), mouse anti-human CD1a antibody (to define iDCs [17]; 1:100 dilution; Zhongshan Golden Bridge Biotech Co., Ltd, Beijing, China), CD3 (Zhongshan Golden Bridge Biotech Co., Ltd, ready to use, Beijing, China) and CD8 (Zhongshan Golden Bridge Biotech Co., Ltd, ready to use, Beijing, China) was carried out on formalin-fixed paraffin, with a 4-μm-thick serial section of tissue, according to the manufacturer’s recommended protocol. The expression of galectin-9 was scored based on staining intensity and percentage. Staining intensity was subclassified as follows: 0, negative; 1, weak; 2, moderate; and 3, strong. The immunohistochemical staining score is equal to the sum of the staining intensity multiplied by the percentage.

The levels of DCs, CD3 + and CD8 + T cells were assessed via the average of 5 count fields per patient in the original magnification of X400 on light microscopy. The grading of the immunostaining was performed in a blinded manner through the consensus of two experienced pathologists.

### MMR status

IHC for four MMR proteins (MLH1, PMS2, MSH2 and MSH6) was performed on formalin‐fixed, paraffin‐embedded tissue obtained from representative sections of the resection specimens. MLH-1 (Ventana 790–5091), PMS2 (Ventana 790–5094), MSH2 (Ventana 790–5093), and MSH6 (Ventana 790–5092) were performed according to previously validated protocols using the Ventana auto-staining platform (Roche). Normal colonic crypt epithelium adjacent to the tumor, lymphoid cells and stromal cells served as internal positive controls. The cases with loss of any of MMR protein expression were defined as dMMR, and the cases without loss of MMR protein expression were defined as pMMR (Supplement Fig).

### Analysis of publicly available databases

#### LinkedOmics database analysis

The LinkedOmics database (http://www.linkedomics. org/login.php) is a web-based platform for analyzing 32 TCGA cancer-associated multidimensional datasets [18]. Galectin-9 mRNA expression was statistically analyzed using Pearson’s correlation coefficient. The function module of LinkedOmics performs analysis of Gene Ontology biological process (GO_BP) and KEGG pathways by gene set enrichment analysis (GSEA). The rank criterion was FDR < 0.05, and 1000 simulations were performed.

#### TIMER database analysis

TIMER is a comprehensive resource for systematic analysis of immune infiltrates across 32 cancer types from TCGA (https://cistrome.shinyapps.io/timer/) [19]. We analyzed the correlation of galectin-9 expression with the abundance of immune infiltrates, including CD4 + T cells, CD8 + T cells, Th1 cells, DCs, and NK cells, as well as tumor purity. The correlation of galectin-9 expression with various immune cell-related genes was examined.

#### Tumor-immune system interactions database analysis (TISIDB)

TISIDB is a web portal for evaluating the interaction of tumor and immune systems that integrates multiple heterogeneous data types (http://cis.hku.hk/TISIDB/index.php) [20]. To further determine the relationship between galectin-9 mRNA expression and the abundance of immune infiltrates, TISIDB was used to determine the correlation between galectin-9 mRNA expression and tumor-infiltrating lymphocytes (TILs).

### Statistical analysis

Statistical analyses were performed with the unpaired Student’s t test or one-way analysis of variance when comparing two groups and ANOVA when comparing more than two groups. To determine correlations between galectin-9 expression and CD208 + DCs, CD1a + DCs, CD3 + T cells and CD8 + T cells, Pearson’s correlation test was used. Student’s t test, Fisher’s exact test, the χ2 test, and Spearman correlation analysis were performed with *p* < 0.05 as a significance level. P and R values were calculated based on the analysis of Pearson’s correlation. All statistical analyses were performed with GraphPad Prism 7.0 software.

## Result

### Patient characteristics

Of the 139 participants, 84 (60.43%) were male and 55 (39.57%) were female. The average age was 57 years, ranging from 20 to 84 years. The clinicopathological data of the CRC patients are summarized in the Supplement Table. All cases were divided into two groups based on tumor location and MMR expression. There were 60 cases (43.17%) in the right-sided group and 79 cases (56.83%) in the left-sided group. For MMR expression, there were 71 cases (51.08%) in the dMMR group and 68 cases (48.92%) in the pMMR group. The N stage was divided into 3 groups according to the American Joint Cancer Committee (AJCC) staging system as follows: 66 cases (47.48%) were N0, 55 cases were N1 (39.57%), and 18 were N2 (12.95%). In addition, we observed vascular invasion in 17 cases (12.2%), perineural invasion in 7 cases (5.0%) and tumor budding in 9 cases (6.5%).

### Galectin-9 expression was reduced and correlated with MMR status in CRC

**Fig. 1:**
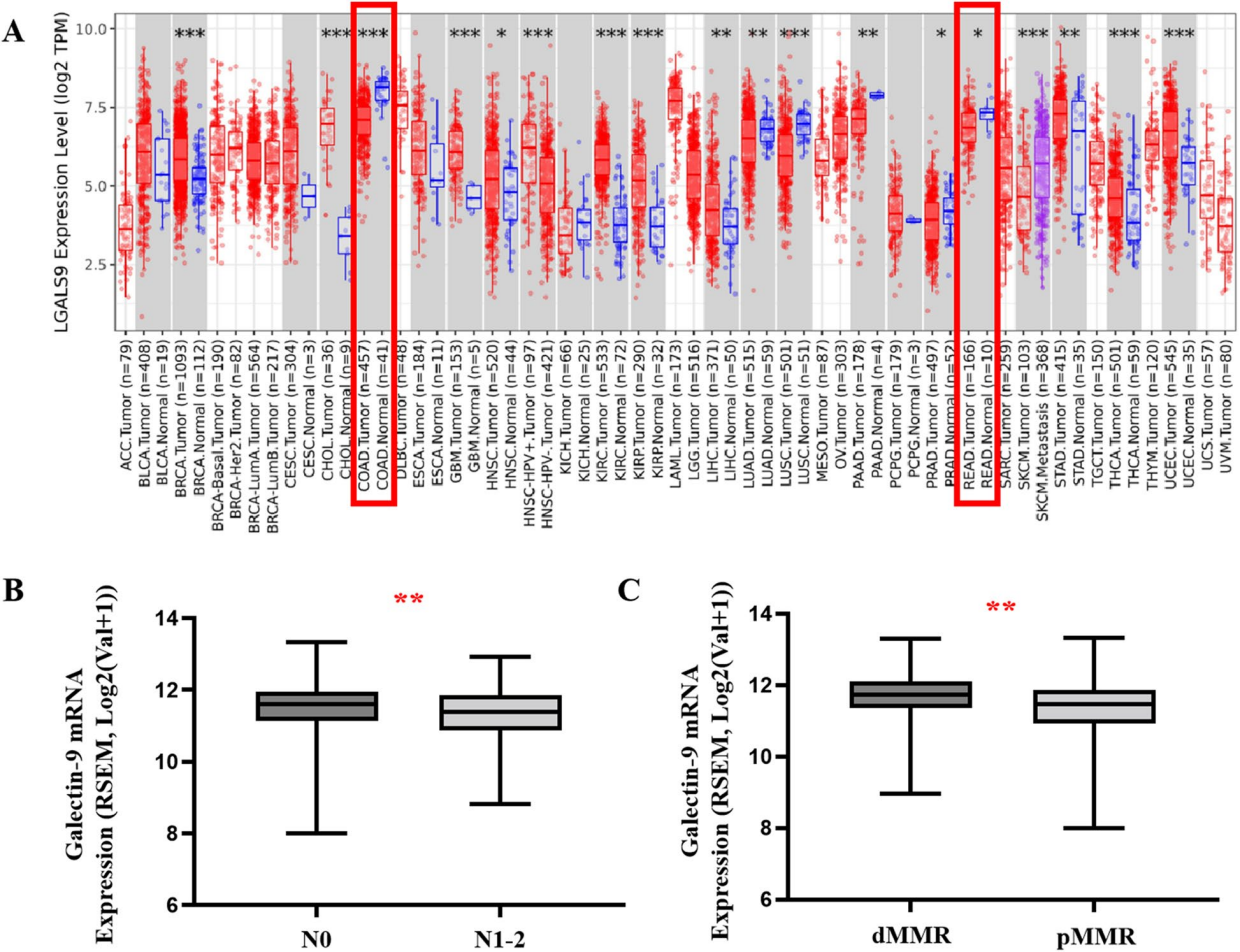
Galectin-9 expression in CRC patients based on TCGA. **A** The expression levels of LGALS9 in different tumor types in the TCGA database (TIMER); distributions of gene expression levels are displayed using box plots. The statistical significance computed by the Wilcoxon test is indicated by the number of stars (*: *p* < 0.05; **: *p* < 0.01; ***: *p* < 0.001). The red box indicates the expression difference of galectin-9 in tumor tissue and adjacent tissue in colon (*N* = 451) and rectal cancer (*N* = 166); **B** Boxplot showing galectin-9 transcription in N0 and N1-2 samples from TCGA (LinkedOmics; *N* = 375), ** *p* < 0.01; (C) Boxplot showing galectin-9 transcription in dMMR and pMMR samples from TCGA (LinkedOmics; *N* = 375), ** *p* < 0.01

As shown in Fig. 1A, the analysis of TCGA RNA-seq data using the TIMER database showed that galectin-9 mRNA expression was significantly downregulated in CRC compared with normal tissues.Then, we downloaded data from the LinkedOmics Database to analyze the relationship between galectin-9 mRNA (LGALS9) expression and clinicopathological factors. With the increase in N stage, the expression of galecin-9 decreased significantly (mean: 11.50 vs. 11.30; *p* = 0.0096, Fig. 1.B). In addition, the expression level of galectin-9 mRNA in dMMR patients was significantly higher than that in pMMR patients (*p* = 0.0011; Fig. 1C).

**Fig. 2:**
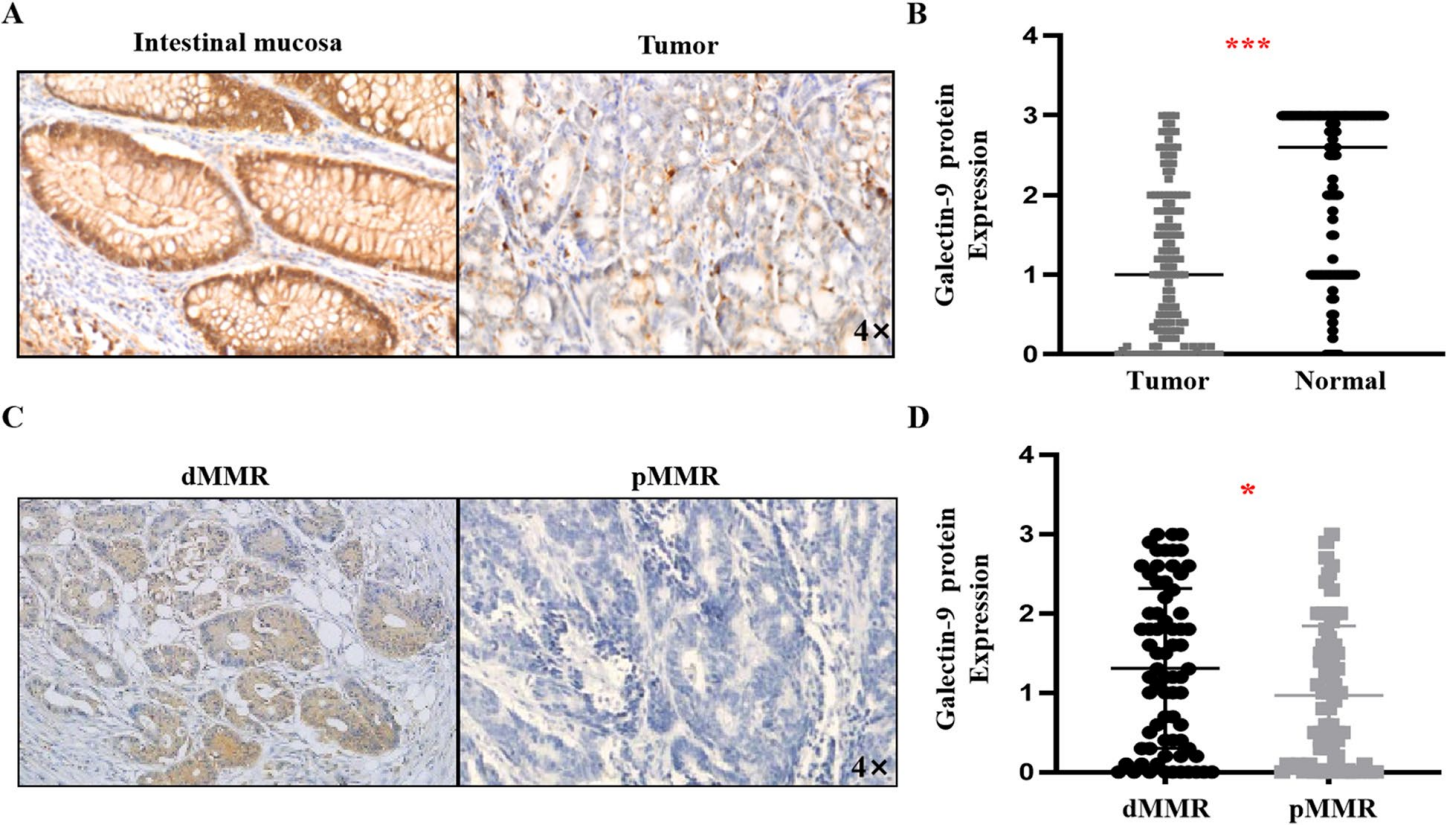
Galectin-9 expression in CRC patients based on tissue samples. **A** Immunohistochemistry of galectin-9 staining in matched normal mucosa (left) and CRC tumor tissue (right) based on our tissue samples; **B** Scatter plot graph showing the relative expression of galectin-9 in matched normal mucosa and CRC tumor tissue (*N* = 113); *** *p* < 0.001. **C** Representative IHC staining images of galectin-9 in CRC patients with dMMR and pMMR; **D** Scatter plot showing the relative expression of galectin-9 in CRC patients with different MMR statuses (*N* = 139), * *p* < 0.05

To further verify the results of the bioinformatics analysis, CRC tissue samples were analyzed. Galectin-9 protein expression was also significantly reduced in CRC tumor tissue (Fig. 2A and B), which is consistent with the results of a previous study^14^. In 56 (82.3%) of 68 patients with pMMR and 61 (85.9%) of 71 patients with dMMR, galectin-9 positivity was observed. Galectin-9 expression was higher in dMMR tumors than in pMMR tumors (mean: 1.311 vs. 0.968; *p* = 0.0335, Fig. 2C and 2D). In addition, the high expression of galectin-9 is also related to the right colon (mean: 1.348 (right) vs. 0.989 (left); *p* = 0.0274), earlier T stage (mean: 1.838(T1-2) vs. 1.072(T3-4); *p* = 0.0054), N stage (mean: 1.422(N0) vs. 0.892(N1-2); *p* = 0.0009) and TNM stage (mean: 1.442(I-II) vs. 0.8918(III-IV); *p* = 0.0009) but not to vascular invasion (mean:1.176(positive) vs. 1.139(negative); *p* = 0.8797), perineural invasion (mean: 1.629(positive) vs. 1.118(negative); *p* = 0.1679) or tumor budding (mean: 1.289(positive) vs. 1.133(negative); *p* = 0.6378).

### CD208 + DC and CD1a + DC infiltration in MMR subtypes in CRC

**Fig. 3:**
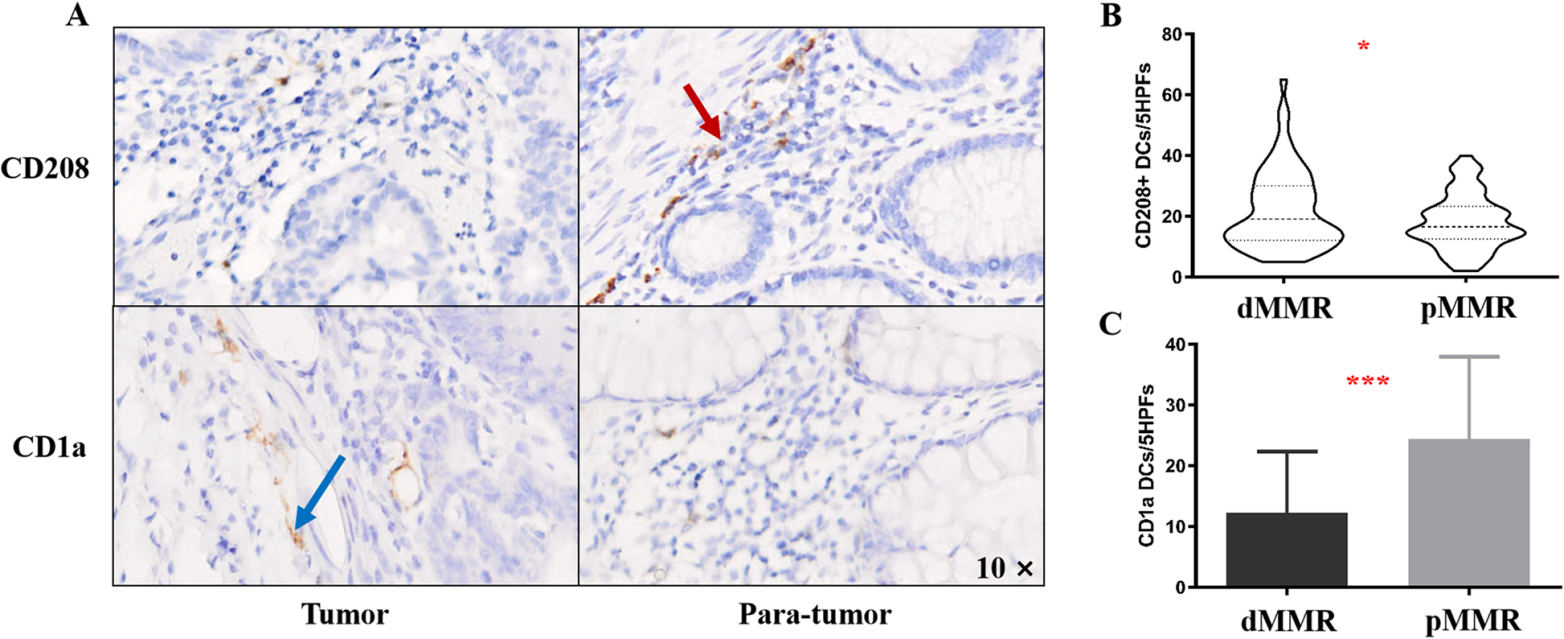
DCs infiltrate the tumor microenvironment of CRC patients. **A** IHC was performed to assess the frequency of CD208 + DCs (red arrow) and CD1a + DCs (blue arrow). **B** Violin plot showing the frequency of CD208 + DCs in CRC patients with different MMR statuses, * *p* < 0.05. **C** Column bar graph showing the frequency of CD1a + DCs in CRC patients with different MMR statuses, *** *p* < 0.001

As depicted in Fig. 3A, CD208 + DCs were detectable in all CRC samples at various frequencies (20.24 ± 11.34 DCs/5 HPFs), which often accumulated along the invasive tumor margin and were preferentially located in the tumor stroma. The number of CD208 + DC cells around the normal mucosa was significantly greater than that around the tumor tissue (*p* < 0.0001). Among 75 patients, the level of CD1a + DC cell infiltration was evaluated. In contrast to CD208 + DCs, CD1a + DCs in CRC tissues were significantly higher than those in normal tissues (*p* < 0.0001). These results suggest that CD208 + DCs are a component of the immune microenvironment and may contribute to the regulation of antitumor immune responses in CRC.

Next, we investigated whether the CD208 + DC counts differed based on different clinicopathological factors. The invasion of CD208 + DCs decreased significantly with increasing T stage (mean: 28.15(T1-2) vs. 19.22 (T3-4); *p* = 0.0062) and N stage (mean: 22.85 (N0) vs. 17.53(N1-2); *p* = 0.0052). However, no statistically significant differences were observed for M stage (mean: 14.20 (M0) vs. 20.28 (M1); *p* = 0.2389), although M1 stage patients exhibited a tendency toward a lower number of CD208 + DCs compared with M0 stage patients, which may be related to the lower number of M1 stage patients. For CD208 + DC populations, higher cell densities were related to the dMMR phenotype than to the pMMR phenotype (mean: 22.20vs. 17.82;*p* = 0.0219, Fig. 3B). A similar phenomenon was also observed in the comparison of the right and left colon, but no significant difference was found (mean: 18.62 (right) vs. 21.95 (left); *p* = 0.0851). In addition, CD208 + DCs infiltration was not associated with vascular invasion (mean:20.44(positive) vs. 17.29(negative); *p* = 0.2831), perineural invasion (mean:20.00(positive) vs. 21.14(negative); *p* = 0.7952) or tumor budding (mean:19.84(positive) vs. 23.22(negative); *p* = 0.3866). A dense CD1a + DC infiltrate was observed in pMMR tumors (mean: 12.21(dMMR) vs. 24.39 (pMMR); *p* < 0.0001, Fig. 3C).Unfortunately, the association of CD208 + DCs with T stage (mean: 15.00(T1-2) vs. 17.17(T3-4); *p* = 0.7819), N stage (mean: 16.88(N0) vs. 17.18(N1-2); *p* = 0.9261), M stage (mean: 16.97 (M0) vs. 19.00 (M1); *p* = 0.7663) and tumor location (mean: 15.38 (right) vs. 17.98 (left); *p* = 0.4197) did not reach statistical significance.

### Galectin-9 expression is correlated with antigen processing and presentation pathways and mDC infiltration in CRC

To further explore the relationship between galectin-9 mRNA expression and immune regulation, we performed GO functional annotation and GSEA through the LinkedOmics platform. High expression of galectin-9 mRNA was closely correlated with antigen processing and presentation (NES = 2.378, *p* = 0). GO biological process analysis showed that galectin-9 mRNA was involved in interferon-gamma (normalized enrichment score (NES) = 2.688, *p* = 0), response to type I interferon (NES = 2.5667, *p* = 0), adaptive immune response (NES = 2.3495, *p* = 0), and antigen processing and presentation (NES = 2.3313, *p* = 0).

Then, the expression levels of antigen presentation-related genes in tumor tissues of dMMR and pMMR patients based on TCGA data were analyzed. The expression levels of *B2M(p* = *2.926e-05), HLA-C(p* = *2.628e-03), HLA-E(p* = *5.5166e-08), HLA-F(p* = *9.376e-03), TAP1(p* = *1.972e-09), TAP2(p* = *1.245e-04)* and *TAPBP (p* = *2.652e-12)*in tumor tissues of dMMR patients were significantly higher than those of pMMR patients. This result suggests that there is a difference in the antigen presentation ability between dMMR and pMMR patients. We next analyzed the correlation between galectin-9 expression and antigen presentation-related genes in CRC patients. The results via the TIMER database showed that galectin-9 expression was positively correlated with *HLA-A, HLA-B, HLA-C, HLA-E, HLA-F, B2M, TAP1, TAP2,* and *TAPBP* gene expression (Table 1). These data strongly suggest that *LGALS9* is closely related to antigen presentation in CRC.

**Table 1:** The correlation between galectin-9 mRNA expression and MHC-I pathway antigen presentation-related gene mRNA expression based on TIMER analysis

Gene	COAD				READ			
	NONO		Purity		NONO		Purity	
	Cor	*p*	Cor	*p*	Cor	*p*	Cor	*p*
*B2M*	0.259	1.79E-08	0.215	1.27E-05	0.293	1.30E-04	0.237	5.00E-03
*HLA-A*	0.271	3.77E-09	0.269	3.59E-08	0.365	1.29E-06	0.38	3.96E-06
*HLA-B*	0.384	1.66E-17	0.372	9.34E-15	0.402	7.77E-08	0.383	3.23E-06
*HLA-C*	0.305	2.66E-11	0.294	1.49E-09	0.357	2.35E-06	0.357	1.58E-05
*HLA-E*	0.452	2.02E-24	0.424	4.17E-19	0.423	1.88E-08	0.433	9.98E-08
*HLA-F*	0.395	0	0.38	2.32E-15	0.471	2.20E-10	0.467	6.76E-09
*TAP1*	0.359	2.18E-15	0.349	4.46E-13	0.352	3.33E-06	0.308	2.27E-04
*TAP2*	0.349	1.35E-14	0.322	2.83E-11	0.288	1.73E-04	0.279	8.95E-04
*TAPBP*	0.354	6.03E-15	0.336	3.42E-12	0.317	3.19E-05	0.363	1.12E-05

Abbr: *COAD* Colon adenocarcinoma, *READ* Rectum adenocarcinoma

**Fig. 4:**
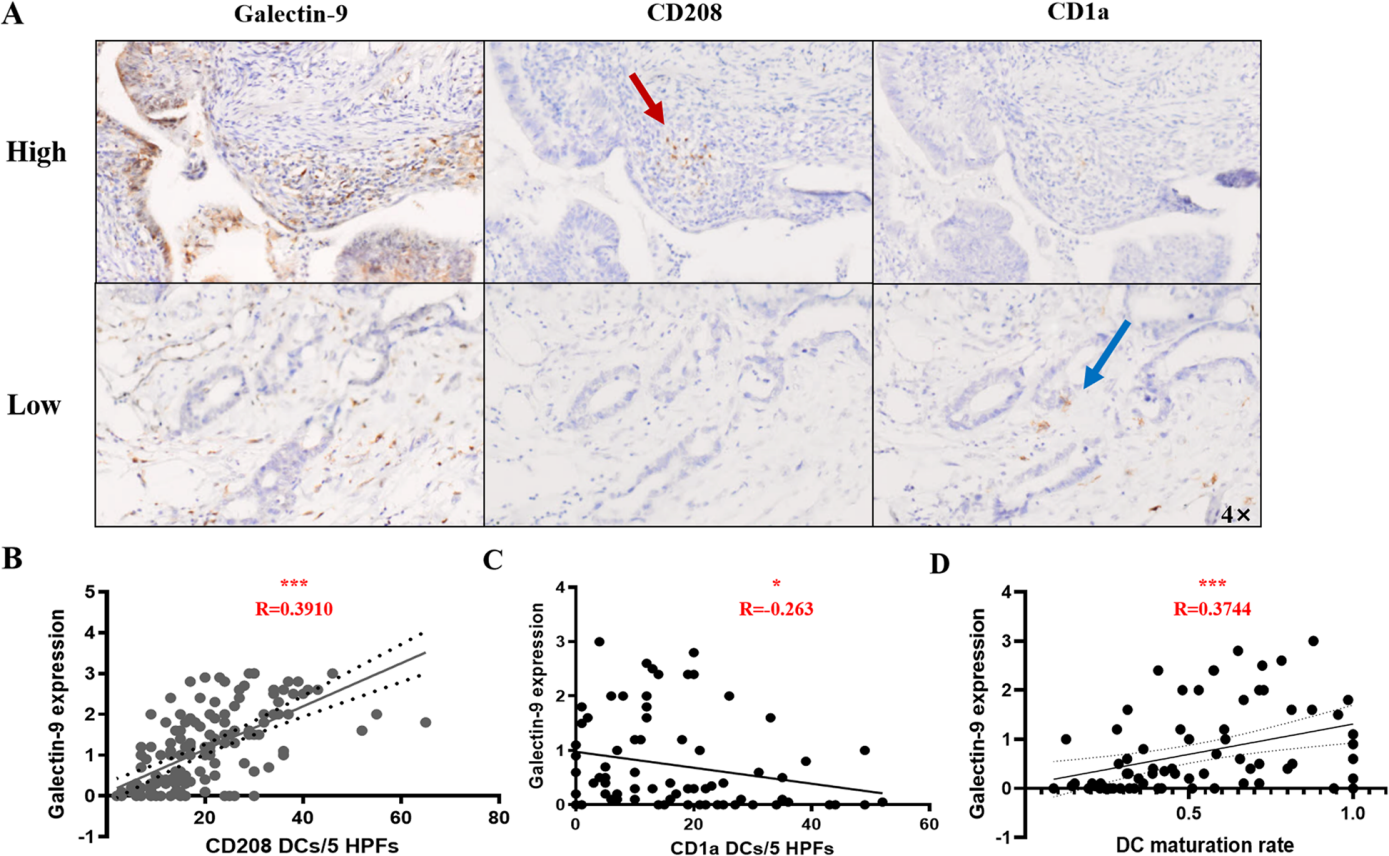
Correlation between galectin-9 expression and DC infiltration. **A** Representative IHC images of CD208 (red arrow) and CD1a (blue arrow) obtained from a patient with high galectin-9 expression and a patient with low galectin-9 expression. **B** Linear analysis of galectin-9 expression and CD208 + DC infiltration (*N* = 139), ***p < 0.001; **C** Linear analysis of galectin-9 expression and CD1a + DC infiltration (*N* = 75), * *p* < 0. (D) Linear analysis of galectin-9 expression and DC maturity rate (*N* = 75), * *p* < 0.05

Next, we investigated the relationship between galectin-9 expression and DC infiltration. Interestingly, tumors with high expression of galectin-9 had more infiltration of CD208 + DCs but less infiltration of CD1a + DCs (Fig. 4A). Galectin-9 expression exhibited a high correlation with CD208 expression (*R* = 0.3910, *p* < 0.001; Fig. 4B). CD1a expression decreased with the increased expression of galectin-9 (*R* = -0.263, *p* = 0.042; Fig. 4C). The correlation between galectin-9 expression and DC maturity rate was also analyzed. The equation used for calculating DC maturity is as follows: DC maturity rate = CD208 + DCs/(CD208 + DCs plus CD1a + DCs). Galectin-9 expression exhibited a linear positive correlation with the DC maturation rate (*R* = 0.3744, *p* = 0.0009; Fig. 4D). The DC maturation rate was higher in dMMR patients than in pMMR patients (*p* = 0.0001).

### Galectin-9 expression is associated with reduced T-cell-mediated immune activation in CRC TMEs

TILs are considered to be prognostic indicators for CRC [21]. The associations between galectin-9 mRNA expression and the level of TIL infiltration in CRC were analyzed via the TIMER database. After correlation adjustments by purity, the findings demonstrated that a positive correlation exists between the galectin-9 mRNA expression level and infiltrating levels of CD4 + T cells (*R* = 0.17, *p* = 4.79e-03), CD8 + T cells (*R* = 0.225, *p* = 1.63e-04), DCs (R = 0.324, *p* = 3.90e-08) and NK cells (*R* = 0.324, *p* = 3.90e-08) in colon cancer, although no statistically significant difference was found in rectal cancer. In addition, to further verify the role of galectin-9 in the TME, we also examined the relationship between the expression levels of galectin-9 and immunological infiltration in the TISIDB database. Spearman’s correlation analysis illustrated that galectin-9 was strongly related to immune infiltration in COAD and READ (Table 2), especially for the five most significant infiltrators of immune cells, as follows: activated CD8 T cells (*r* = 0.231 in COAD, *r* = 0.276 in READ), central memory CD4 T cells (*r* = 0.203 in COAD, *r* = 0.138 in READ), CD56 bright NK cells (*r* = 0.216 in COAD, *r* = 0.245 in READ), activated DCs (*r* = 0.218 in COAD, *r* = 0.296 in READ), and type-1 T helper (Th1) cells (*R* = 0.116 in COAD, *R* = 0.219 in READ).

**Table 2:** Correlation between galectin-9 expression and the infiltration of various immune cell types in CRC tumors based on TISIDB analysis

Immune cell type	COAD		READ	
	R	*P*	R	*P*
Activited CD8 T cell	0.231	6.31E-07	0.276	3.24E-04
Centrol memory CD8 T cell	0.194	2.84E-05	0.143	6.56E-02
Effector memory CD8 T cell	0.206	9.38E-06	0.227	3.26E-03
Centrol memory CD4 T cell	0.203	1.23E-05	0.138	7.59E-02
Th1	0.116	1.26E-02	0.219	4.56E-03
NK	0.142	2.36E-03	0.108	1.65E-01
CD56 bright NK cell	0.216	3.27E-06	0.245	1.44E-03
CD56 dim NK cell	0.373	7.27E-17	0.271	4.14E-04
Activited DC	0.218	2.66E-06	0.296	1.11E-04

Abbr: *COAD* Colon adenocarcinoma, *READ* Rectum adenocarcinoma

**Fig. 5:**
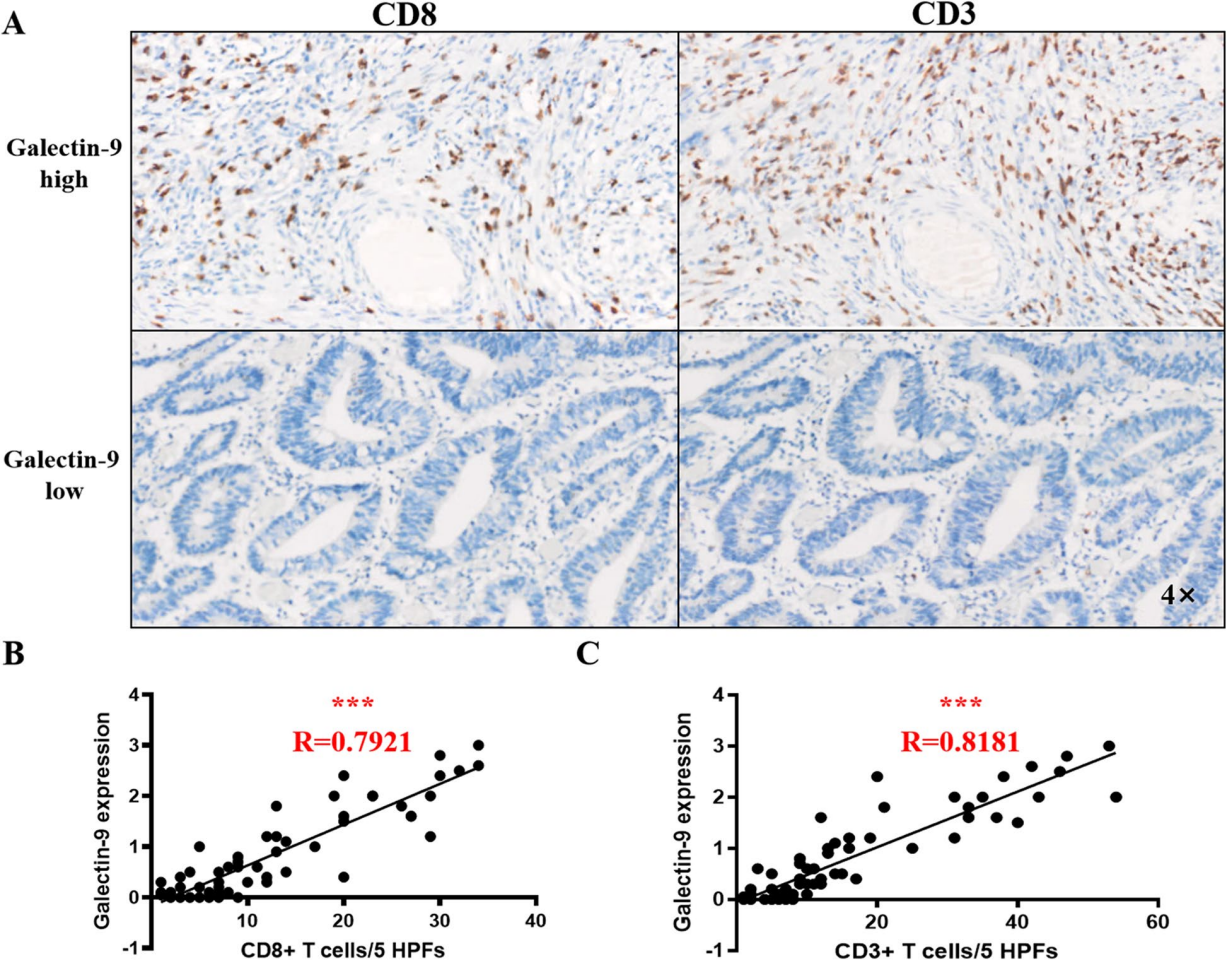
Correlation between galectin-9 expression and CD3 + T-cell and CD8 + T-cell infiltration. **A** Representative IHC images of CD3 + and CD8 + cells obtained from a patient with high galectin-9 expression and a patient with low galectin-9 expression. **B** Linear analysis of galectin-9 expression and CD8 + T-cell infiltration (*N* = 62), *** *p* < 0.001; **C** Linear analysis of galectin-9 expression and CD3 + T-cell infiltration (*N* = 62), *** *p* < 0.001

To examine the effects of galectin-9 expression on the human TME, we performed IHC for CD8 and CD3 in CRC patient tissue sections. Consistent with the previous results, patients with low galectin-9 expression tended to have fewer CD8 + cells and CD3 + cells than patients with high galectin-9 expression. The intensity of galectin-9 in the tumor was positively correlated with CD8 + cells and CD3 + cells, indicating that high galectin-9 expression may control the number of T cells in the TME (Fig. 5).

To further determine the correlations between galectin-9 and TILs, we analyzed the relationship between galectin-9 and marker genes of different immune cells in COAD and READ via GEPIA databases. After correlation adjustments by purity, the expression levels of CD4 + T cells, CD8 + T cells, NK cells, Th1 cells and DCs presented strong correlations with galectin-9 expression in COAD and READ (Table 3).

**Table 3:** Correlation analysis of galectin-9 and the expression of various immune cell gene markers in CRC tumors based on GEPIA

Immune cell type	Gene markers	CRC	
		R	*P*
T Cell	*CD3D*	0.3	7.20E-09
	*CD3E*	0.25	1.20E-06
	*CD3G*	0.28	5.60E-08
	*CD2*	0.28	6.70E-08
CD4 + Tcell	*CD4*	0.21	4.20E-05
CD8 + Tcell	*CD8A*	0.22	2.50E-05
	*CD8B*	0.006	9.10E-01
	*TBX21*	0.23	1.20E-05
	*EOMES*	0.14	7.80E-03
	*LCK*	0.035	5.10E-01
	*IFNG*	0.29	1.10E-08
	*PRF1*	-0.014	7.80E-01
	*GZMA*	0.34	3.80E-11
	*GZMB*	-0.12	2.10E-02
	*GZMH*	0.21	7.10E-05
	*GZMK*	0.11	4.10E-02
	*GZMM*	0.24	4.40E-06
	*CXCL9*	0.23	9.00E-06
	*CXCL10*	0.23	6.90E-06
Th1	*IFNG*	0.29	1.10E-08
	*TBX21*	0.23	1.20E-05
	*TNF*	0.1	5.00E-02
	*STAT4*	0.19	2.90E-04
	*STAT1*	0.37	4.40E-13
NK cell	*KIR3DL1*	0.17	1.40E-03
	*KIR3DL2*	0.19	2.00E-04
	*KIR3DL3*	0.14	8.40E-03
	*KIR2DS4*	0.083	1.10E-01
	*KIR2DL1*	0.1	5.60E-02
	*KIR2DL3*	0.12	1.80E-02
	*KIR2DL4*	0.25	1.10E-06
DC	*BDCA-1(CD1C)*	0.083	1.10E-01
	*BDCA-2(CLEC4C)*	0.12	2.20E-02
	*BDCA-4(NRP1)*	0.037	4.90E-01
	*CD11b(ITGAM)*	0.1	5.50E-02
	*CD11c(ITGAX)*	0.1	5.20E-02
	*HLA-DPB1*	0.26	7.30E-07
	*HLA-DQB1*	0.17	1.00E-03
	*HLA-DRA*	0.29	1.30E-08
	*HLA-DPA1*	0.28	4.90E-08
	*CD208(LAMP3)*	0.32	2.00E-10

## Discussion

In this study, the correlation of galectin-9 expression with the main clinicopathological factors (MMR status) was analyzed by bioinformatics and tissue sample analysis. MMR status was significantly associated with antigen presentation- and processing-related cells and molecules. We also validated the significant relationship between galectin-9 expression and the previously established DC and T-cell activation-related factors, antigen-presenting cells, tumor infiltrating lymphocytes, and DC markers (CD208 as mDC and CD1a as imDC).

The expression and prognostic value of galectin-9 seems to be tumor type dependent. Some studies demonstrate a reduced expression compared with normal tissue, with the opposite results in other cancers. A detailed summary of the differential expression levels of galectin-9 in various tumors was provided previously [13]. A complicating factor is the fact that galectin-9 mRNA transcripts undergo extensive splicing and generate different galectin-9 isoforms [22]. For example, it was reported that the mRNA level in gastric cancer was decreased more than the normal tissue decrease [23], while the protein levels were increased [24]. Unfortunately, no antibodies have been described to distinguish between these isoforms. These results limit the immunohistochemical analysis to the evaluation of the total galectin-9 expression level. In our previous [14] and current research, both the galectin-9 mRNA and protein expression were significantly decreased when comparing tumor tissue with normal mucosa. Researchers have studied the prognostic significance of galectin-9 in cancers and revealed conflicting results depending on cancer types. The decrease in galectin-9 expression suggests poor clinicopathological factors and prognosis in CRC patient tissue [14]. Patients with galectin-9 expression had a longer survival time than those with negative lesions in hepatocellular carcinoma [25]. Two meta-analyses reported that higher galectin-9 expression in solid cancer tissue is associated with improved cancer-specific survival (CSS) [26] or overall survival (OS) [27]. In addition, the subgroup analysis revealed a significant relationship between higher galectin-9 expression and both CSS and OS in digestive cancers, which further supports our conclusion. Overall, the number of studies was limited and the sample size was small in some studies. Therefore, further research is required to obtain more definitive evidence in larger patient groups.

Antigen processing and presentation are important for the immune and therapeutic susceptibility of tumors [7]. DCs support antitumor immunity by stimulating CD8 + T-cell responses. Combinatorial approaches addressing DCs and T cells represent an attractive strategy to achieve higher response rates across patients. However, mDCs often accumulate less in the TME and fail to provoke effective antigen presentation and T-cell stimulation. Many studies indicate that galectin-9 plays a suppressive role in T lymphocyte activation, but its function in the antigen presentation process is not well understood. An association between the expression of galectins and antigen presentation has been reported. For example, galectin-8 was reported to favor immobilized antigen extraction and presentation in vitro [28]. In addition, it also activates DCs and stimulates antigen-specific immune response elicitation [29]. Galectin-1 regulates DC differentiation, signaling and migration via the extracellular matrix [30–32]. However, Galectin-3 could interfere with DC fate determination, regulating apoptosis in T lymphocytes and inhibiting B-lymphocyte differentiation into immunoglobulin-secreting plasma cells [33]. Galectin-9 induces the maturation of human monocyte-derived dendritic cells [34]. Galectin-9 administration increased the number of Tim-3 + CD86 + mDCs and enhanced antitumor immunity [35]. Recently, intracellular galectin-9 was shown to be indispensable for plasma membrane integrity and structure in DCs and essential for C-type lectin receptor-mediated pathogen uptake by DCs [36]. In our study, high galectin-9 expression was positively correlated with a large amount of mDC infiltration and negatively correlated with imDC infiltration in CRC, and galectin-9 was involved in interferon-gamma, response to type I interferon, adaptive immune response, and antigen processing and presentation. The above results suggest that galectin-9 is involved in the regulation of the antitumor immune response affecting mDCs.

TMEs infiltrating antigen-specific T cells have been described as T-cell-inflamed environments. These environments include variable numbers of CD8 + T cells, which are markers of susceptibility to immunotherapy. In contrast, tumors with non-T-cell infiltrating TMEs do not clinically benefit from immunotherapy. The identification of molecular markers associated with a non-T-cell-infiltrating TME is therefore necessary and important for the development of combinational immunotherapy strategies. In response to our findings, the galectin-9 expression level in the TMEs of human CRC tissues was strongly positively associated with CD8 + T-cell infiltration, suggesting that low galectin-9 expression in the tumor stroma could be used to identify non-T-cell-inflamed TMEs, which are less responsive to immunotherapy.

## Conclusions

In this study, we found that a higher expression of galectin-9 was significantly related to dMMR and right-sided location. Moreover, galectin-9 was significantly associated with DC maturation, activation and the T-cell immune response. The high expression of galectin-9 shows richer infiltration of mature DCs and CD8 + T cells. In summary, galectin-9 expression can be a good prognostic marker and could guide future immune combination therapies in CRC patients with pMMR.

## Supplementary Information

**Additional file 1:** **Supplement Fig.** Immunohistochemical graph of mismatch repair proteins using continuous section.

**Additional file 2:** **Supplement Table.** Summary of clinicopathological data of the enrolled cases.

## Data Availability

The data generated in this study are available upon request from the corresponding author.
