# Supplementary material for: Galectin-9 expression clinically associated with mature dendritic cells infiltration and T cell immune response in colorectal cancer
Source: BMC Cancer. 2022 Dec 16;22:1319. doi: 10.1186/s12885-022-10435-4 (PMC9756675; doi:10.1186/s12885-022-10435-4)
Supplement: Supplementary file 1 — Additional file 1: Supplement Fig. Immunohistochemical graph of mismatch repair proteins using continuous section. [file 12885_2022_10435_MOESM1_ESM.docx]

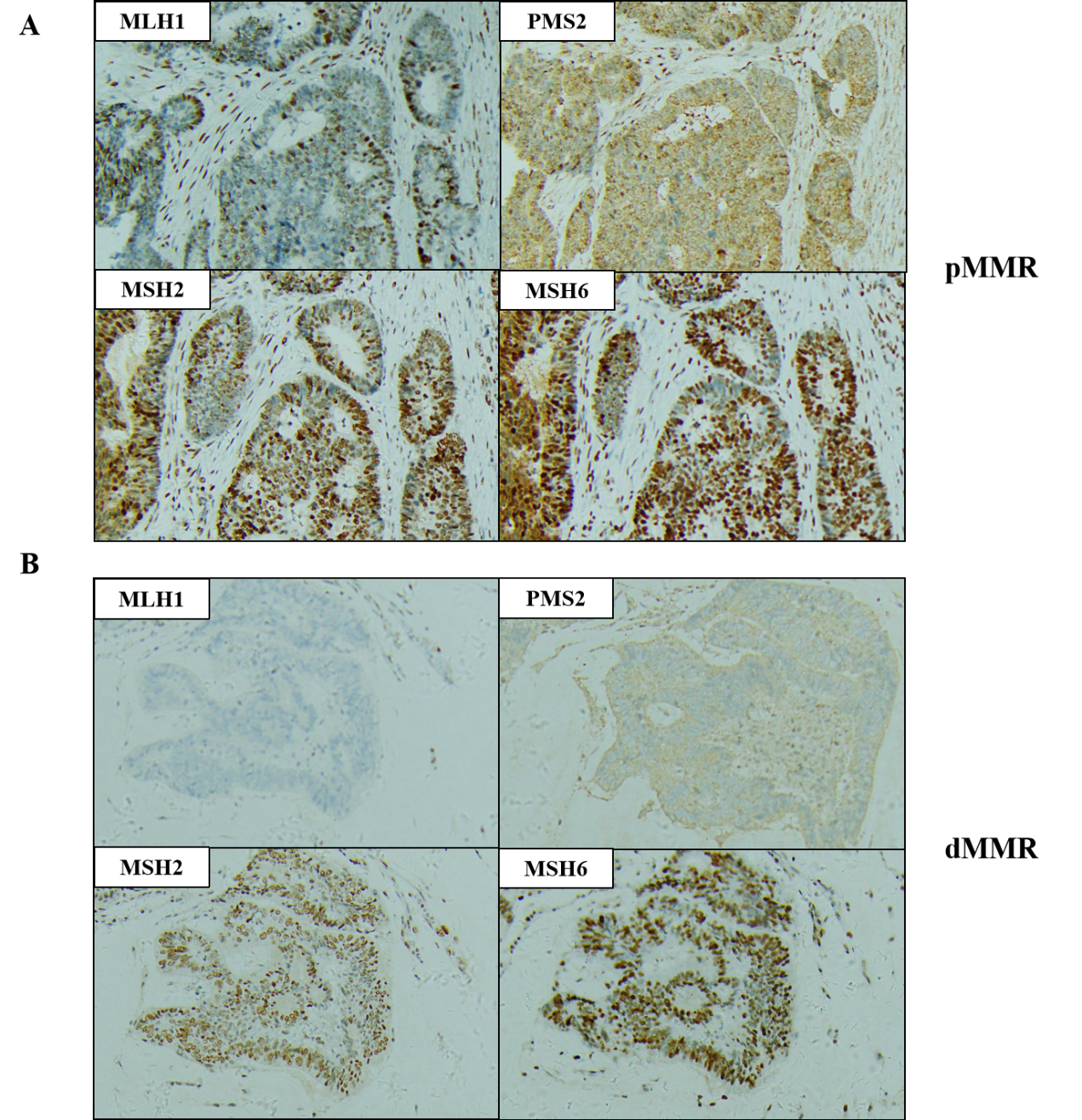


**Supplement Fig Immunohistochemical graph of mismatch repair proteins using continuous section.** (A) a representative graph of pMMR CRC patients; immunophenotype: MLH1(+), PMS2(+), MSH2(+) and MSH6(+); (B) a representative graph of dMMR CRC patients; immunophenotype: MLH1(-), PMS2(-), MSH2(+) and MSH6(+).
