# Supplementary material for: Galectin-9 expression clinically associated with mature dendritic cells infiltration and T cell immune response in colorectal cancer
Source: BMC Cancer. 2022 Dec 16;22:1319. doi: 10.1186/s12885-022-10435-4 (PMC9756675; doi:10.1186/s12885-022-10435-4)
Supplement: Supplementary file 2 — Additional file 2: Supplement Table. Summary of clinicopathological data of the enrolled cases. [file 12885_2022_10435_MOESM2_ESM.docx]

| Supplement Table Summary of clinicopathological data of the enrolled cases. | | |
| --- | --- | --- |
| Clinicopathological Parameters | N | （%） |
| Age (yrs) | (Range)  20-84 | (mean±sd)  57±13.98 |
| Gender |  |  |
| Female | 55 | 39.57% |
| Male | 84 | 60.43% |
| location |  |  |
| Left | 79 | 56.83% |
| Right | 60 | 43.17% |
| MMR |  |  |
| dMMR | 71 | 51.08% |
| pMMR | 68 | 48.92% |
| T stage |  |  |
| T1 | 3 | 2.16% |
| T2 | 10 | 7.19% |
| T3 | 101 | 72.66% |
| T4 | 25 | 17.99% |
| N stage |  |  |
| N0 | 66 | 47.48% |
| N1 | 55 | 39.57% |
| N2 | 18 | 12.95% |
| M stage |  |  |
| M0 | 134 | 96.40% |
| M1 | 5 | 3.60% |
| TNM stage |  |  |
| I | 11 | 7.91% |
| II | 55 | 39.57% |
| III | 68 | 48.92% |
| IV | 5 | 3.60% |
| Vascular invasion | 17 | 12.23% |
| Perineural invasion | 7 | 5.04% |
| Tumor budding | 9 | 6.47% |
